# The immunity function of rodlet cells in the intestine of Binni fish (*Mesopotamichthys sharpeyi*)

**DOI:** 10.5455/javar.2022.i594

**Published:** 2022-06-27

**Authors:** Abdulkarim Jafar Karim, Ali Hussein Hassan, Khalid Hadi Kadhim, Khalid Kamil Kadhim

**Affiliations:** 1 Department of Anatomy, College of Veterinary Medicine, University of Baghdad, Baghdad, Iraq; 2 College of Dentistry, University of Sulaimani, Sulaymaniyah, Iraq; 3 Department of Anatomy, College of Veterinary Medicine, University of Al-Muthanna, Al-Muthanna, Iraq

**Keywords:** Fish, immunohistochemical, intestine, Mesopotamichthys sharpeyi, rodlet

## Abstract

**Objective:**

Rodlet cells produce secretions of glycoproteins in nature. This study investigated the microscopic morphology, histochemical and immunohistochemical reactions, and distribution of the rodlet cells in the gut of Binni fish (Mesopotamichthys sharpeyi).

**Materials and Methods:**

Thirty samples were obtained from the cranial, middle, and caudal portions of Binni intestine immediately after being euthanized, fixed in Bouin’s solution for 18 h at 24°C, and had undergone routine histological processing, different conventional histochemical stains, and immunostaining with TNF-α and S100 protein antibody.

**Results:**

The intestine of Binni fish showed different stages of rodlet cells classified into three distinctive forms: vesicular, granular, and mature cells. Rodlet cells are poorly stained with hematoxylin and eosin. Their secretory granules have a weak positive reaction with periodic acid-Schiff (PAS) and Alcian blue (AB), and react positively to combined AB and PAS. Rodlet cells were stained lightly with Safranin O, observed pink in color by Giemsa stain, and showed reactivity to Masson’s and Mallory trichrome stains. Rodlet cells were immunostained positively against TNF-α and S100 antibodies, indicating that they have an immune function.

**Conclusions:**

Rodlet cells, with their neutral glycoprotein secretions, play a crucial role in the immunity of Binni fish intestine.

## Introduction

Rodlet cells produce glycoprotein secretions associated with the epithelium and/or the endothelium of the digestive tract [1], pancreas [2], bulbus arteriosus [3], kidney [4], gonads [5], skin [6], operculum epithelium, gill, and gill raker [7], among the mesothelial cells in the abdominal cavity of both freshwater and marine teleost fishes [1]. We encountered various tissues of the same species, but not in all individuals, with particular abundance and size in the intestines of eels [2]. Their name is derived from the large rod-shaped cytoplasmic granules they contain. The rodlet cells are found in bony healthy and parasitized fishes and are occasionally reported in vertebrates other than fishes [8]. Rodlet cells represent normal cells with a normal nucleus, a capsule consisting of filaments, possibly of a contractile nature, and their relationship with adjoining cells [4].

Water quality mainly affects the fish’s respiratory and gastrointestinal tracts [9]. The intestinal mucosa, mainly the hindgut, acts as an essential antigen transport site and modulates the immunity of the gut by involving several substances in the host–parasite interaction [10]. During embryonic development, the rodlet cells develop between the enterocytes throughout the intestinal epithelium, such as in Gadus morhua [11], turbot, gilthead seabream, and common dentex larvae [12]. They are commonly present within both the columnar and stratified epithelial layers. They contain neutral mucus to protect the surface epithelium of the intestine [13]. Histoarchitecture of the liver parenchyma and the biliary mucosa revealed that the rodlet cells are present in black molly and absent in southern platyfish [14–16].

Rodlet cell has been classified as a protozoan parasite since Thèlohan discovered it in 1892 and named it *Rhabdospora thelohani*. Although the parasite theory of origin has fallen out for the past 20 years and is regarded as being of endogenous origin, its exact organ of origin is still questionable. Some investigators argue that the parasite concept is still valid [5,17]. Rodlets could be a form of inclusion body produced in response to infection, while the strong staining reaction of the rodlet cell granules suggests an enzymatic activity [18]. Our study was planned to reveal some criteria of the intestinal rodlet cells in Binni fish (Mesopotamichthys sharpeyi, M. sharpeyi), a native freshwater cyprinid species of the Tigris and Euphrates, Iraq, and investigate the role of rodlet cells in immunity.

## Materials and Methods

### Ethical approval

All procedures were carried out in accordance with the Animal Care and Use Committee, College of Veterinary Medicine, University of Baghdad, and adopted under 2073 on May 18, 2020.

### Animals

This study was conducted using 30 healthy local adult male and female Binni fish (M. sharpeyi), aged 24 ± 3 months and 42.8 ± 0.7 cm long, caught alive from the Al-Forat river in July 2020.

### Histological processing

Immediately after euthanizing the fish, incisions were made through the ventral midline of the fish from just the cranial to the anus to expose the whole intestine. Each specimen was divided into three (cranial, middle, and caudal) regions, washed with 0.9% normal saline solution, and small pieces (1 cm3) from different areas of each region were collected and fixed in Bouin’s solution for 18 h. Routine histological processing was conducted according to Suvarna et al. [19].

### Tissue staining

Staining procedures include hematoxylin and eosin (H&E) to reveal complete histological components. Periodic acid-Schiff (PAS) and Alcian blue (AB) pH 2.5 were used to find out mucopolysaccharides, and combined AB plus PAS staining was used for neutral and acid polysaccharide differentiation. Safranin O (SO), Giemsa, Mallory trichrome (MyT), and Masson’s trichrome (MnT) were used to identify collagen fibers and smooth muscles [19].

### Immunohistochemical technique

Samples were immersed in 0.1 M phosphate-buffered saline (PBS) for 5 min. Kits of primary polyclonal antibodies against TNF-α (code; ab667, supplier; ABCAM^®^, UK) and S100 protein (Ac651 supplier; DAKO^®^, Glostrup, Denmark) were used, and all instructions by the manufacturers were followed. The slides were washed with PBS for 5 min, then placed for 1 h at room temperature with drops of goat anti-rabbit IgG-biotin (1:200; DAKO^®^) as the secondary antibody. After further rinsing, 3,3-diaminobenzidine covered the sections, followed by immersion in nonionized water to stop the reaction, and then, with Mayer’s hematoxylin for 30 sec and washed with PBS [19]. Sections were examined using an Eclipse E200-LED light microscope (NIKON^®^, Japan).

### Statistical analyses

Data were analyzed using one-way analysis of variance in Statistical Package for Social Sciences (SPSS) 16.0 for Windows (SPSS Inc., Chicago, IL). The significance level was selected at *p *< 0.05.

## Results

**Figure 1.**
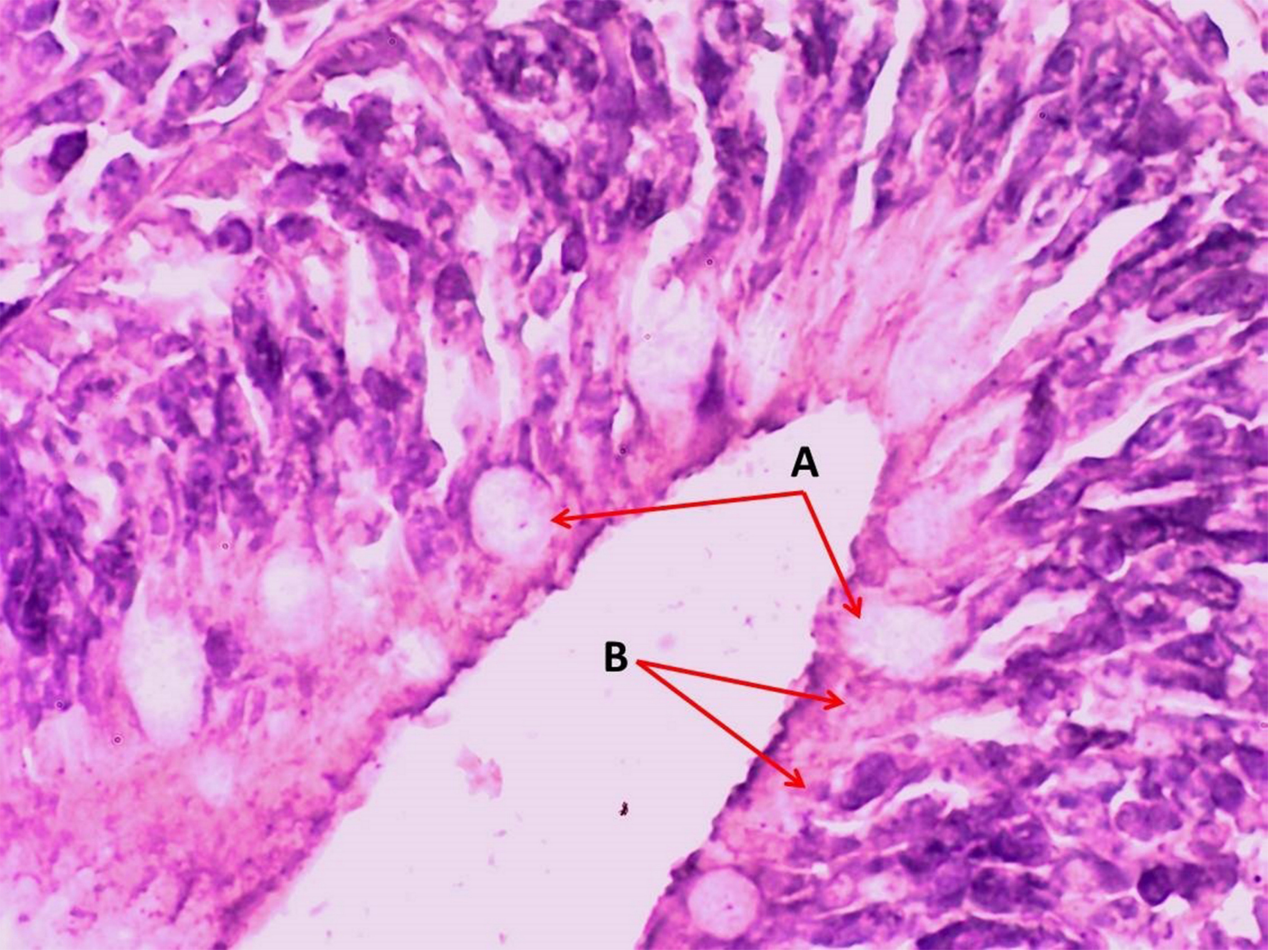
Photomicrograph of the crypt of the caudal portion of the intestine of Binni fish (*M. sharpeyi*). (A) Goblet cells and (B) rodlet cells (H & E stain, ×400).

**Figure 2.**
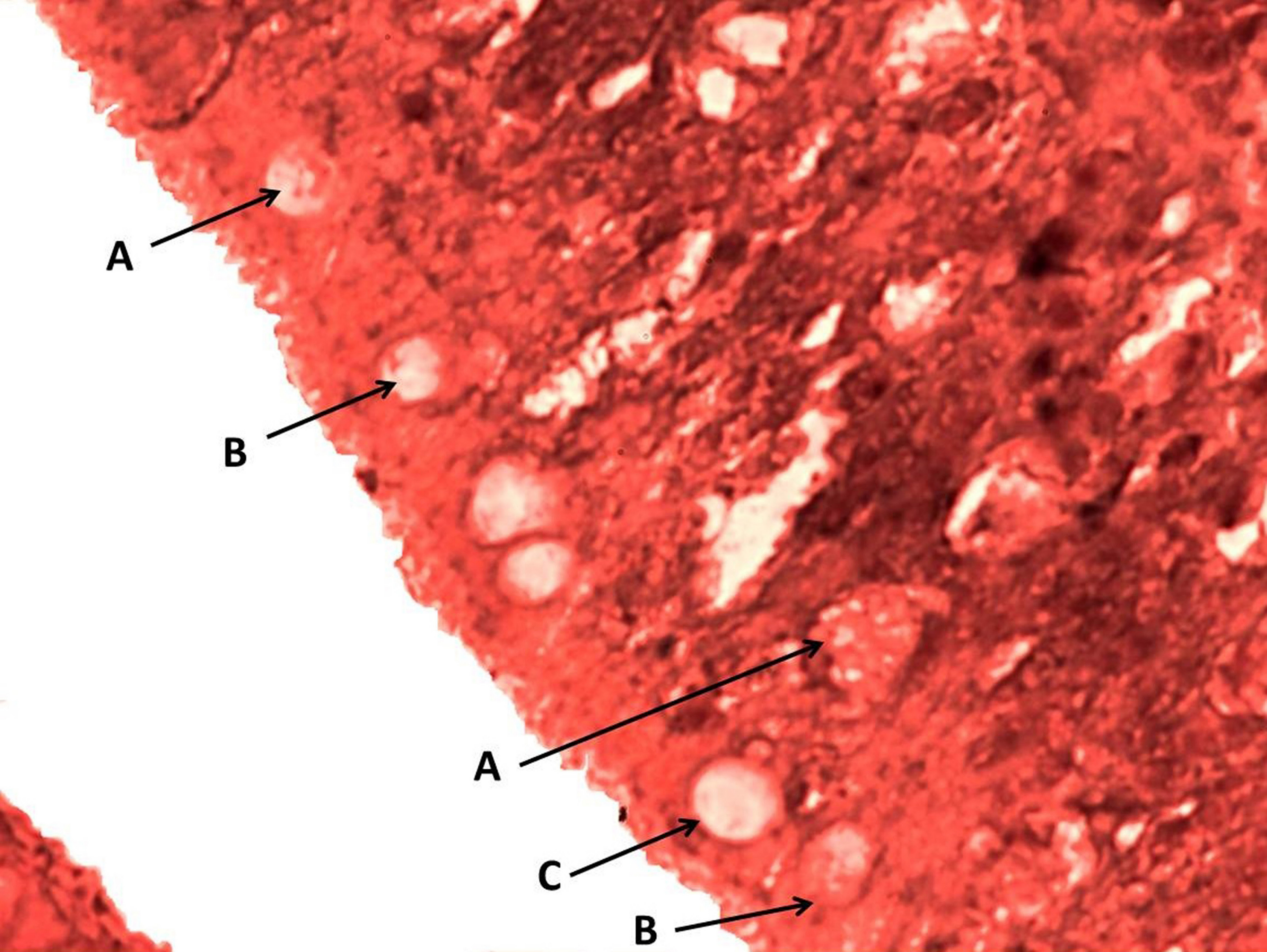
Photomicrograph of the epithelial layer in the middle portion of the intestine of Binni fish (*M. sharpeyi*). (A) Granular rodlet cell, (B) vesicular rodlet cell, and (C) mature rodlet cell (MyT stain, ×400).

Several mature rodlet cells localized to the cranial portion of the intestine showed a significant (p < 0.05) increment comparable with the middle and caudal parts without significant variations between males and females (Table 1). However, with their characteristic features, the goblet cells can be easily differentiated from rodlet cells (Fig. 1). They were mostly observed in different stages in the intestinal epithelium of Binni fish and were classified into three distinctive forms: 1) vesicular cells, 2) granular cells, and 3) mature cells. The vesicular rodlet cell was large, rich in vesicles, and contained extensive cytoplasmic vacuolation with an accumulation of intracellular fibrillar-like components and granules that appeared eosinophilic (Fig. 2). On the other hand, the mature rodlet cell was an elongated bear-shaped cell with a distinct narrow tip. They contain long acidophilic granules filling the cytoplasm and a nucleus at its widest end. These cells become more elongated at their dense central core, forming needle-shaped protrusions into the cell apex (Fig. 2). Granules of the rodlet cells have a dichromatic reaction for glycoprotein staining with a strong affinity. This may indicate that the granules in the basal and apical parts possess different components.

**Table 1.** Number of mature rodlet cells per microscopic field (100 x) in different portions of the intestine of Binni fish (*M. sharpeyi*).

Part of intestine	Number of rodlet cells	
	Male (*n *= 15)	Female (*n *= 15)
Cranial portion	23.77 ± 0.32^Aa^	24.31 ± 0.25^Aa^
Middle portion	18.91 ± 0.27^Ab^	17.82 ± 0.51^Ab^
Caudal portion	10.35 ± 0.41^Ac^	11.16 ± 0.48^Ac^

Data are expressed as mean ± SE.

Different small letters represent a significant difference between rows (*p* ≤ 0.05).

**Figure 3.**
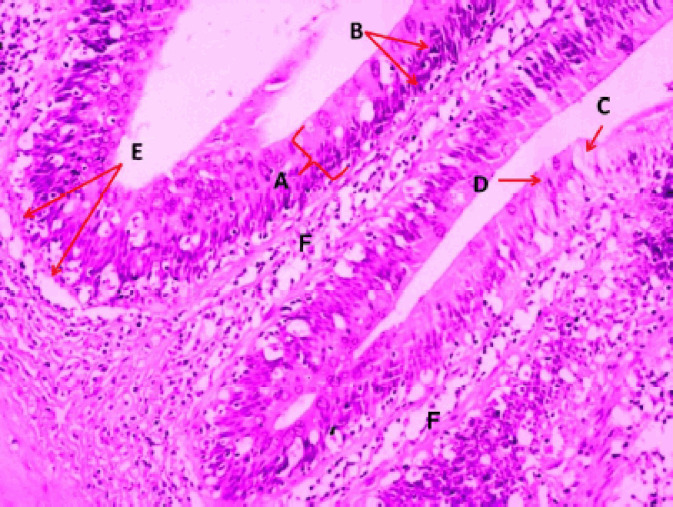
Photomicrograph of the middle portion of the intestine of Binni fish (*M. sharpeyi*). (A) Epithelium, (B) Nuclei of enterocytes, (C) Goblet cell, (D) Mature rodlet cells, (E) Basement membrane, and (F) Lamina propria (PAS stain, ×100).

**Figure 4.**
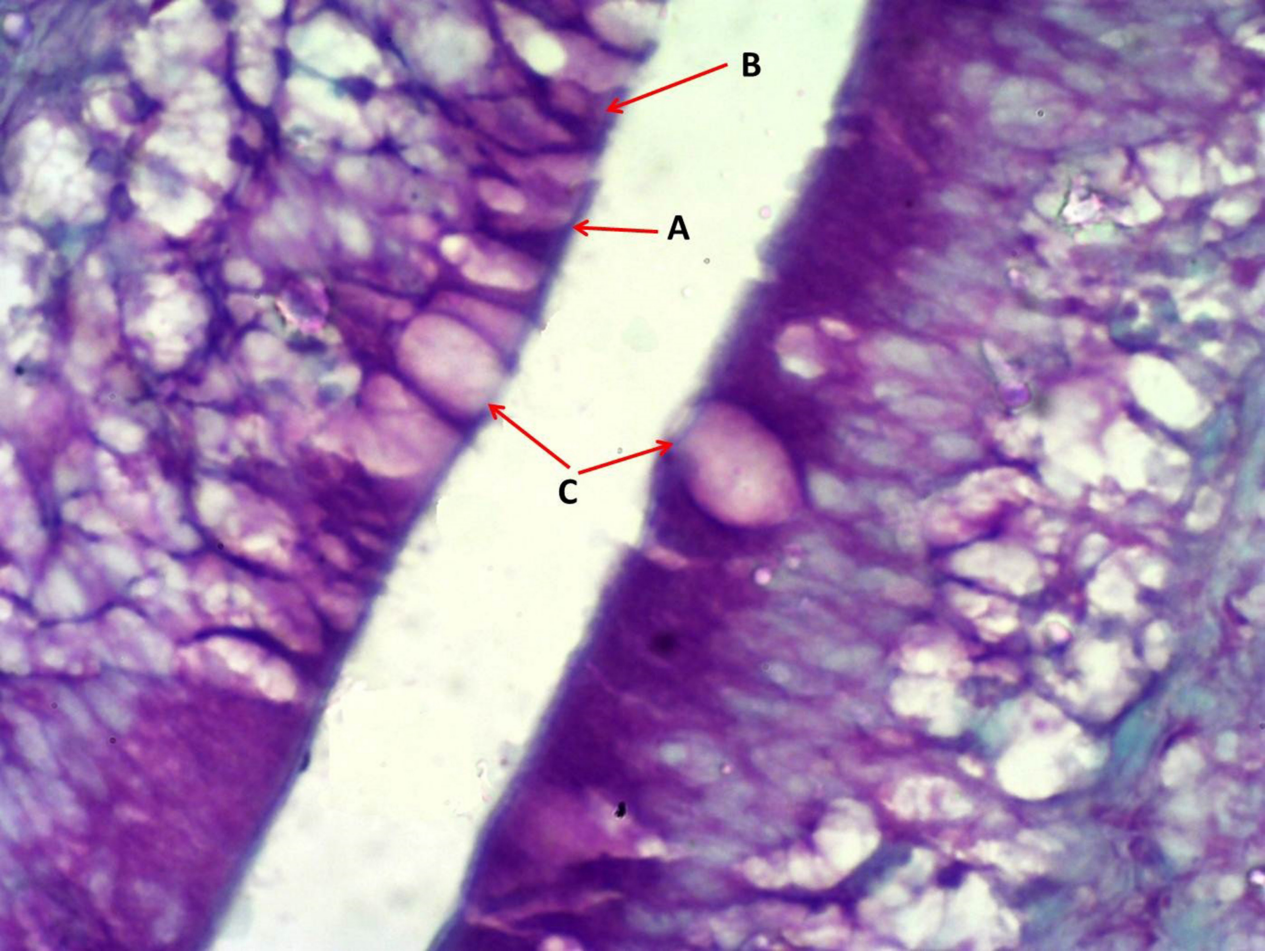
Photomicrograph of the epithelium of the cranial portion of the intestine of Binni fish (*M. sharpeyi*). (A) Granular rodlet cell, (B) Mature rodlet cell, and (C) Goblet cells (combined AB and PAS stain, ×400).

**Figure 5.**
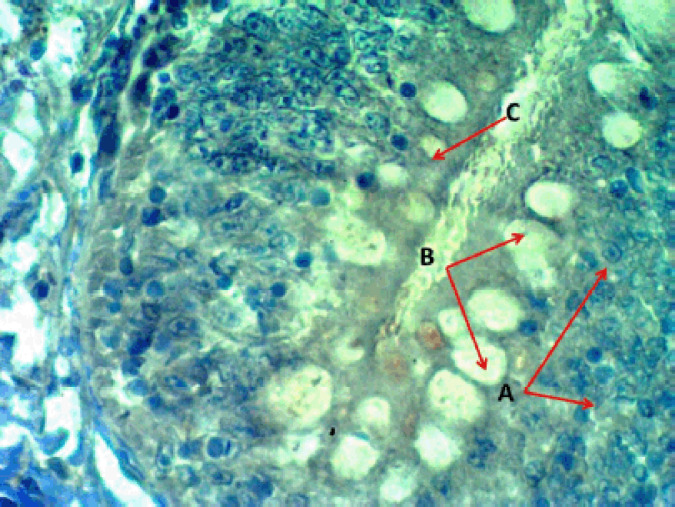
Photomicrograph of the crypt in the cranial portion of the intestine of Binni fish (*M. sharpeyi*). (A) Nucleus of enterocytes, (B) Goblet cells, and (C) Rodlet cell (Masson trichrome stain, ×400).

**Figure 6.**
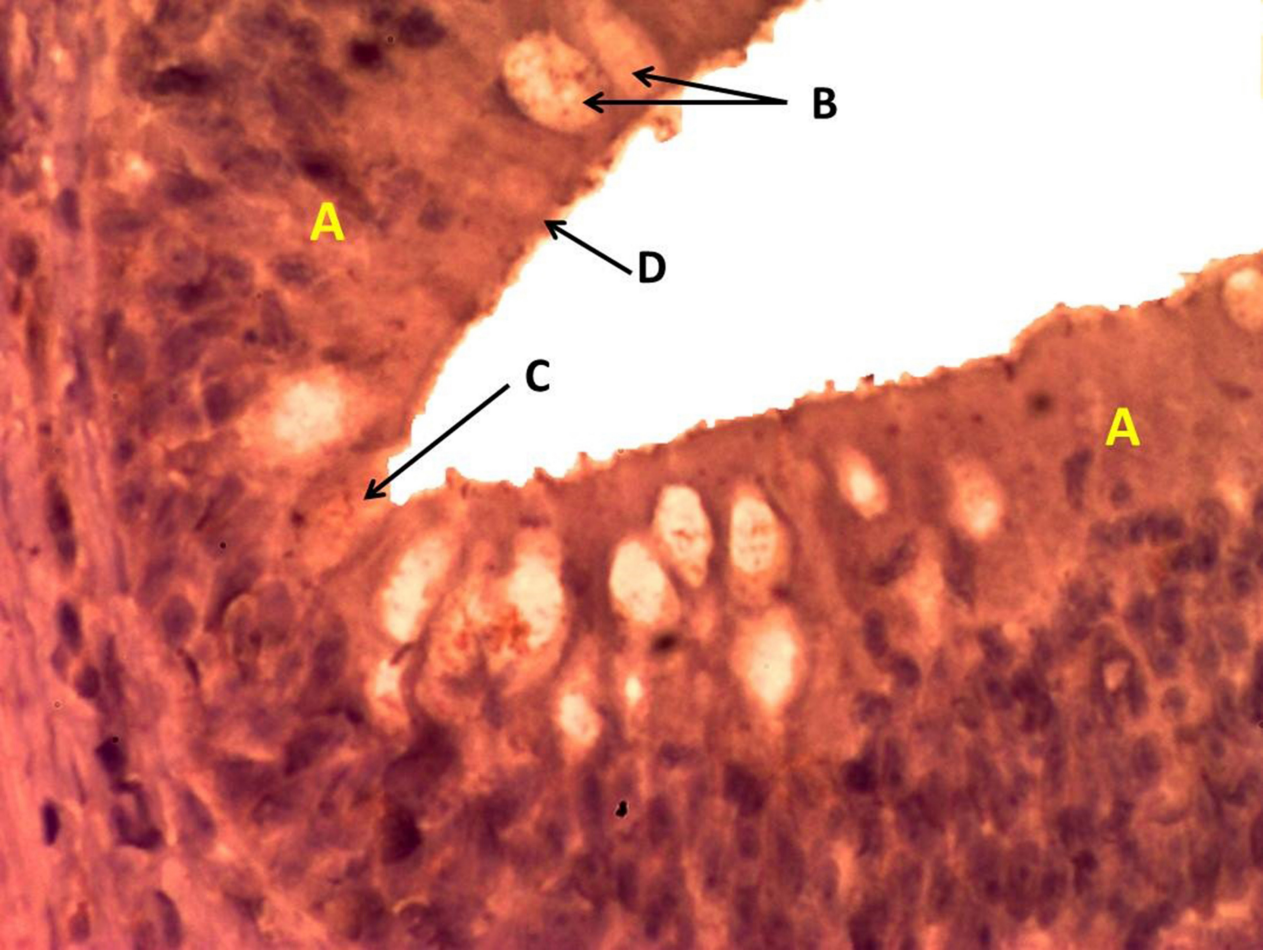
Photomicrograph of the epithelium in the middle portion of the intestine of Binni fish (*M. sharpeyi*). (A) Epithelium, (B) Goblet cells; (C) Vesicular rodlet cell, and (D) Mature rodlet cell (Safranian O stain, ×400).

**Figure 7.**
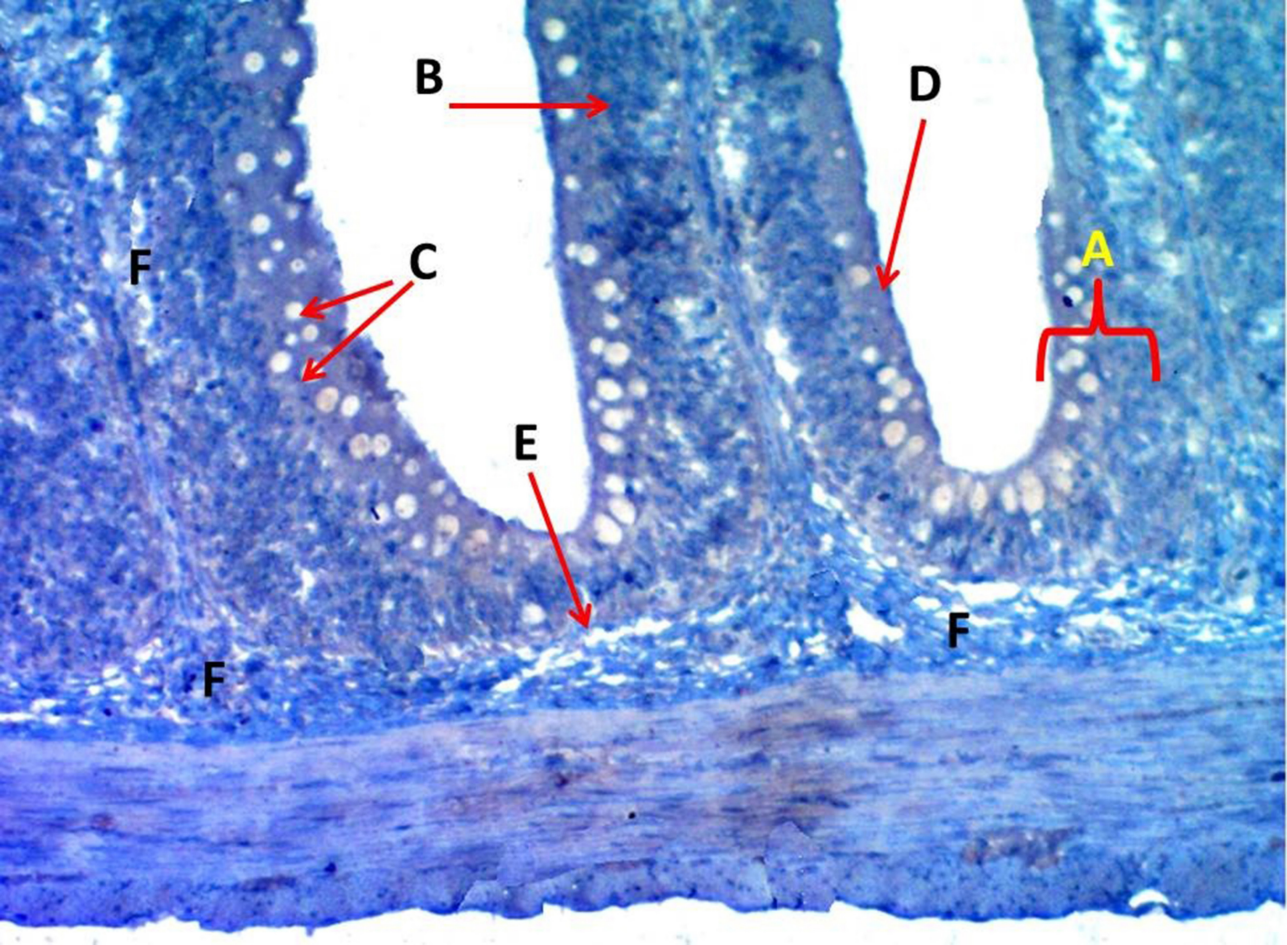
Photomicrograph of villi of the caudal portion of the intestine of Binni fish (*M. sharpeyi*). (A) Epithelium, (B) Nucleus of enterocytes, (C) Goblet cells, (D) Rodlet cell, (E) Basement membrane, and (F) Lamina propria (Masson trichrome stain, ×200).

**Figure 8.**
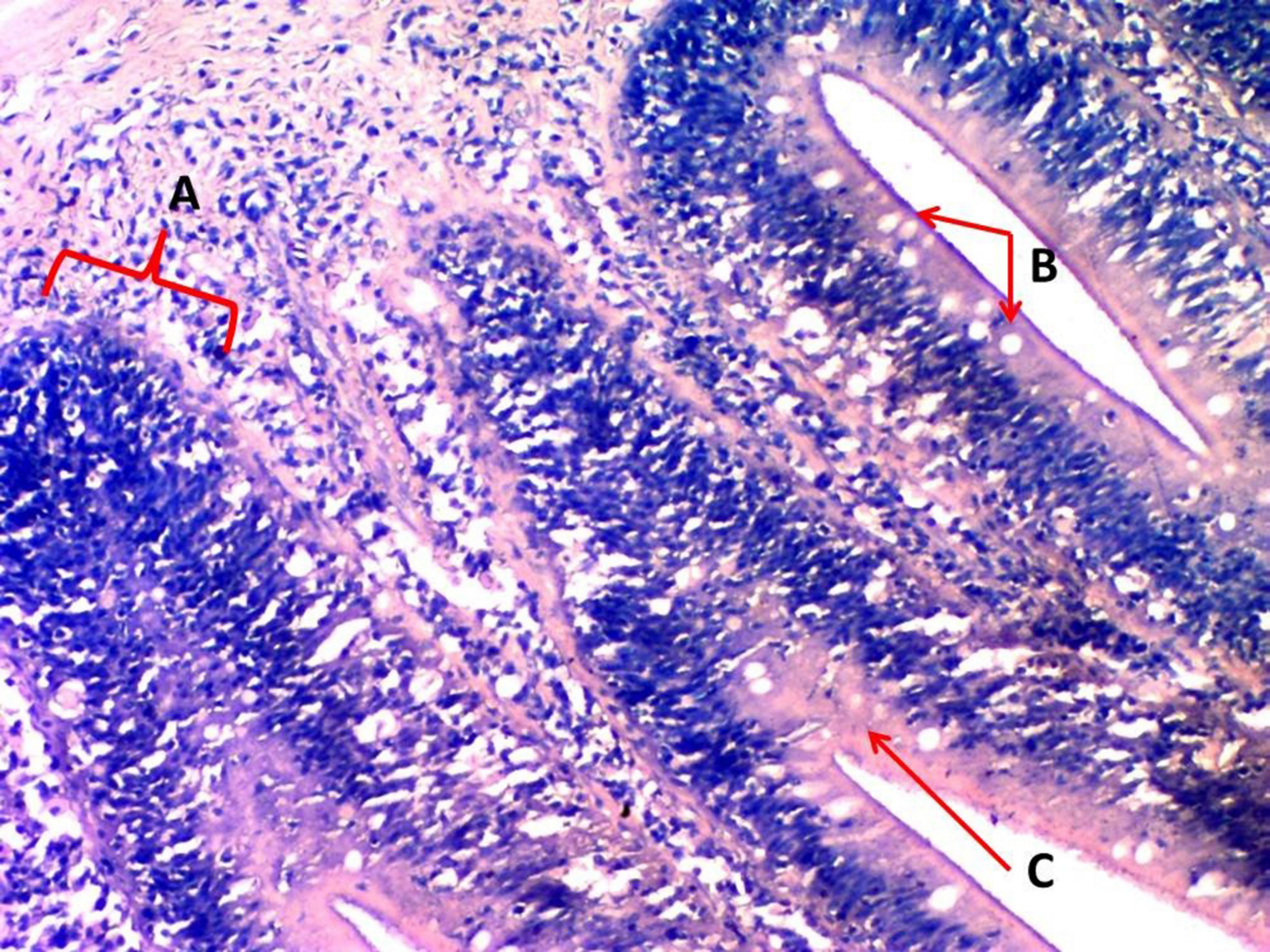
Photomicrograph of the crypt of the middle portion of the intestine of Binni fish (*M. sharpeyi*). (A) Crypts, (B) Goblets, (C) Rodlet cells (AB stain, ×100).

**Figure 9.**
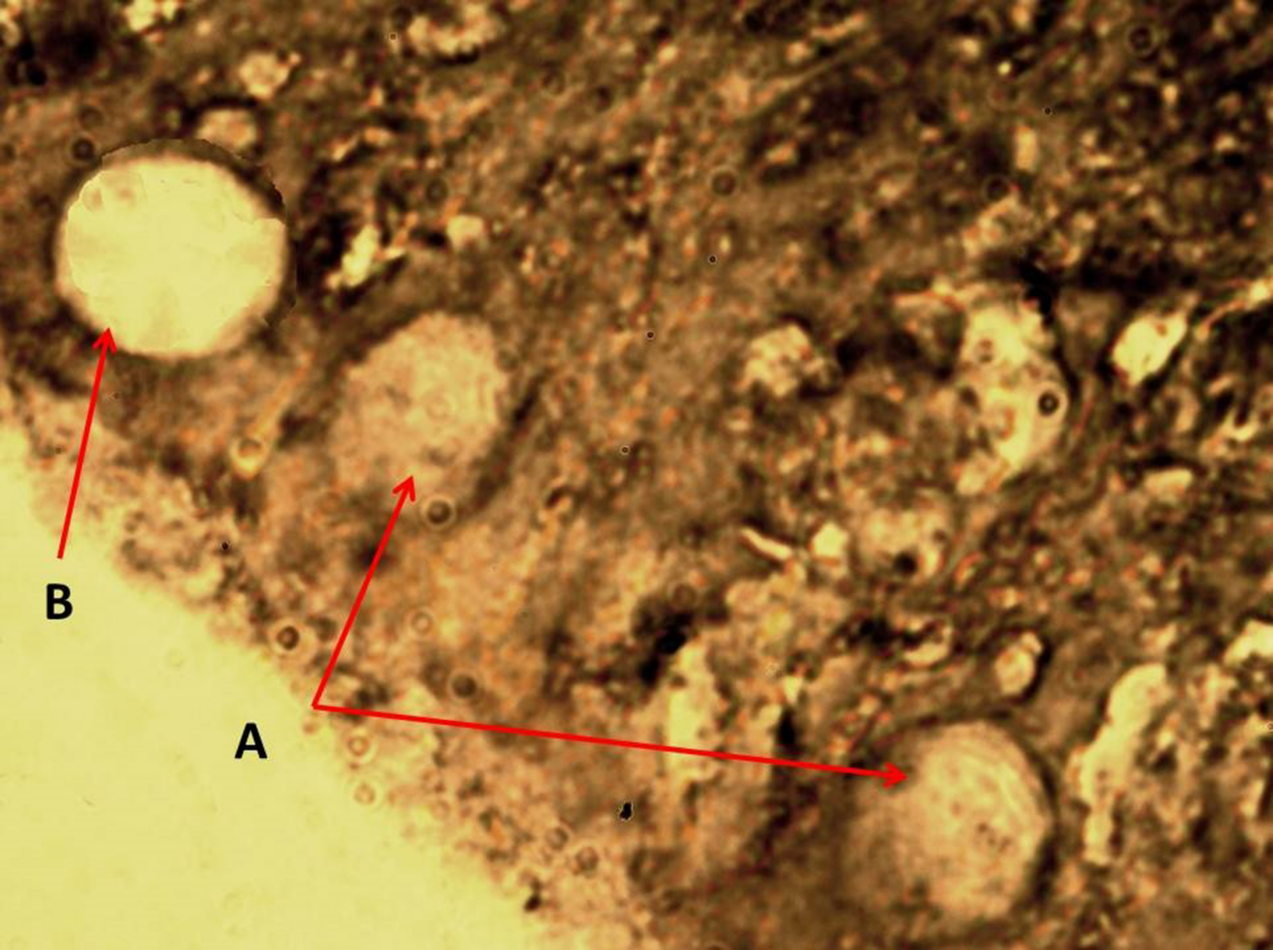
Photomicrograph shows the rodlet cells’ immunoreactivity to S100 protein antibody in the cranial portion of the intestine of Binni fish (*M. sharpeyi*). (A) Granular rodlet cells and (B) Goblet cell (×1,000).

Different capital letters represent a significant difference between rows (*p* ≤ 0.05).

rodlet granules react to PAS staining in all stages (Fig. 3), with different staining reactions to combined AB plus PAS (Fig. 4). Contrary to the mature stage, the vesicular stage had no affinity for it. The MnT exhibited cytoplasmic granulation of rodlet cells (Fig. 5). In the granular and mature stages, rodlet granules reacted to both stains (AB and PAS) (Fig. 4). This variation could be attributed to the different secretions produced by rodlet cells in the different stages. The SO stained the rodlet granules in the vascular and mature stages (Fig. 6). Rodlet cells in the caudal region of the intestine showed similar cytoplasmic granulation (Fig. 7) and did not stain with AB (Fig. 8). TNF-α and S100 protein immunoreactivities were found in mature rodlet cells (Fig. 9).

## Discussion

Standard conventional stains can differentiate rodlet cells into different stages, e.g., H&E, PAS, AB plus PAS, SO, Giemsa, MyT, and MnT. This study showed three distinct phases of rodlet cells in the intestine of Binni fish: vesicular, granular, and mature. This was consistent with Bosi et al. [17], arguing that many rodlet cells can be found in the intestinal epithelium of healthy *Anguilla anguilla*. On the other hand, Abd-Elhafeez and Soliman [20] recorded six categories of endogenous rodlet cells in various organs of ruby shark fish.

Typical rodlet cells were detected 5, 6, 8, and 14 days postfertilization in the intestine, the kidneys, the abdominal cavity among the mesothelial cells, and gills, respectively [1,8]. The endogenous origin of these cells highly supports this. Immunity against enteric helminths in teleost fishes is referred to as the distribution of rodlet cells in the digestive tract [2,11,21]. Their function can be secretory [22,23] or immunological [24]. The proposed defense functions of rodlet cells against pathogens are related to the secretion of piscidin, a peptide with strong antimicrobial activity, and the contractile properties of its fibrous layer [23,25]. The immune function of the rodlet is supported by an increase in the number of rodlet cells in the presence of various chemicals and environmental stressors [18,25]. Rodlet cells are classified as nonspecific immune cells, transport units of genetic material and regulatory elements, and ion transportation cells [7]. Some researchers hypothesized that rodlet cells are in charge of water and electrolyte transport or acting as mucous cells, such as pH control, lubrication, antibiotic effects, and ectoparasite resistance on epithelial surfaces [22]. Rodlet cells represent piscine inflammatory cells that closely resemble mast cells [26]. Piscidin peptides are antimicrobials, as are the protective mechanisms found in the cells of certain fish cells [27].

The histochemical methods revealed numerous rodlet cells in each portion of the intestine with different staining intensities, indicating different amounts of neutral and alkaline mucoglycoproteins. Such secretions aid digestion and emulsification of food into chimes to protect the intestinal epithelium from glycosidases’ deterioration actions and their possible role in osmoregulation [13]. The cell product probably has an enzymatic function similar to the mucin secreted by goblet cells [18,28]. Combined AB plus PAS staining revealed granular rodlet cells with neutral polysaccharides, and there was no reaction to AB because the polysaccharide is not acidic. Some researchers argued a positive peroxidase and alkaline phosphatase in the rodlets’ central core and a peripheral region, suggesting a defensive function against exogenic substances [23,25]. Rodlet granules were positively stained by SO, a cationic dye bound to sulfated glycosaminoglycans, as in the respiratory organs of ruby [20]. Rodlet cells’ reactivity for connective tissue staining by MnT and MyT agrees with Morrison’s [11] study on Pterodoras granulosus.

Similarly, Giemsa was used to stain the rodlet cells. These results are confirmed by Morrison [11]. Rodlet cells, like turbot cells, were immunoreactive to TNF-α [29]. These findings strongly support the inflammatory role of rodlet cells in fishes. Leino [22] assured the presence of actin microfilaments. Accordingly, S100 protein in rodlets may indicate an immune regulatory function in the contractile fibrous layer and rodlet extrusion.

## Conclusion

It is concluded that rodlet cells with their neutral glycoprotein secretions are considered to be normal components of Binni fish intestine. Rodlet cells exert an immunity function.

## List of Abbreviations

AB, Alcian blue; H&E, hematoxylin and eosin; MnT, Masson’s trichrome; MyT, Mallory trichrome; PAS, periodic acid-Schiff; SO, Safranin O.
